# Comparative Evaluation of Orofacial Injuries in Children Playing Roller Skating With and Without Mouthguards: A Non-randomized Controlled Trial

**DOI:** 10.7759/cureus.85671

**Published:** 2025-06-09

**Authors:** Ritesh Kalaskar, Rashmi A Dongarwar, Sandeep Pipare, Shivani Sawant, Anija C. K., Ashita R Kalaskar

**Affiliations:** 1 Pediatric Dentistry, Government Dental College and Hospital, Nagpur, Nagpur, IND; 2 Dentistry, District General Hospital, Chandrapur, Chandrapur, IND; 3 Oral Medicine and Radiology, Government Dental College and Hospital, Nagpur, Nagpur, IND

**Keywords:** athletes, children, conventional mouthguard, orofacial injury, roller skating, tmj pain

## Abstract

Background: Engagement in sports offers substantial physical and psychological benefits for children and adolescents, but also elevates the risk of orofacial injuries such as fractured teeth, soft tissue trauma, and jaw damage. Roller skating, a popular and growing activity among children, presents particular risks due to high speeds and balance challenges. While standard protective gear like helmets, knee pads, and wrist guards are commonly included in skating kits, the use of mouthguards, despite their importance in preventing orofacial injuries, remains limited. Custom-fitted mouthguards made from materials such as ethylene-vinyl acetate offer superior fit, comfort, retention, and protection compared to stock or boil-and-bite types. Key characteristics such as ease of speech and breathing, resistance to tearing, and coverage of teeth, gingiva, and lips make them ideal for young athletes. Parental awareness and involvement are critical factors influencing the adoption of such preventive measures.

Aim: The study aims to evaluate orofacial injuries in 8- to 14-year-old children playing roller skating with or without a mouthguard.

Methods: A non-randomized controlled clinical study was carried out in skating academies across the Nagpur region and at the Department of Pediatric and Preventive Dentistry. Roller skating athletes aged 8 to 14 years were enrolled and divided into two groups, each consisting of 43 skaters. Group A included athletes whose parents consented to both participation in the study and the use of conventional mouthguards during skating; these athletes were provided with 4 mm-thick conventional mouthguards. Group B consisted of athletes whose parents agreed to participation but declined the use of mouthguards for their children; thus, these athletes skated without any mouthguards. Participants were monitored over a one-year period, with incidents of falls and skating-related orofacial injuries recorded throughout the study duration.

Results: Eighty-six athletes (43 in each group) between 8 and 14 years old were included in the study. There were 28 males and 15 female athletes in Group A and 26 males and 17 females in Group B. The prevalence of falls in both groups was 100%. A non-significant difference was observed in the hard and soft dental tissue injuries in permanent teeth (p=0.229) as well as deciduous teeth (p=0.494) between the two groups. Additionally, 4.7% of athletes in Group B suffered from comminution of the alveolar socket. Intraoral soft tissue injuries depicted a statistically significant difference between both groups (p=0.002). No significant difference was observed in the occurrence of extraoral injury and other body parts injury in both groups. Following one year of intervention, a significantly lower number of athletes in Group A reported temporomandibular joint (TMJ) pain compared to Group B, relative to baseline values at the start of the study. Based on the questionnaire evaluation conducted after one year of use, athlete acceptance of the mouthguard was rated as excellent by 19 athletes, good by 21, and moderate by three.

Conclusion: Custom-fitted mouthguards significantly reduce orofacial injuries, especially dental fractures and TMJ pain in children who roller skate, without affecting balance or fall risk. The study supports promoting mouthguard use in pediatric high-risk sports and encourages further education and innovation in design.

## Introduction

Infants, children, and adolescents benefit physically and mentally from sports but face a higher risk of orofacial injuries like chipped teeth, fractures, and soft tissue damage [1]. Falls and collisions can cause some serious orofacial trauma [2]. Traumatic dental injuries have an adverse impact on oral health-related quality of life in children and adolescents injuries in children [3]. Orofacial injuries are a significant concern in sports, particularly in disciplines where the use of protective gear such as mouthguards is not mandated. Unlike contact sports like football and hockey, where mouthguard usage is often compulsory, many popular sports, such as baseball, basketball, and soccer, do not enforce such requirements. As a result, athletes participating in these sports may face a heightened risk of facial trauma. Studies examining injury trends in sports that do not require mouthguards, such as baseball, basketball, and soccer, have found that orofacial injuries account for roughly 3% to 38% of all injuries specific to those sports [4].

Roller sports, particularly roller skating, are popular among children in India, both as a recreational pastime and as a competitive discipline. The activity has been increasingly integrated into urban school curricula and supported by numerous skating clubs in cities and towns. Although recreational skating can improve the health of children through exercise, participation in skating activities exposes children to the risk of orofacial injury due to the combination of high speeds and the potential for losing balance on uneven surfaces, particularly for developing children [5].

Despite the high risk of orofacial injuries in roller skating, protective gear like helmets and pads is widely used, but mouthguards are often neglected. Studies highlight the prevalence of such injuries, with inline skating identified as a leading cause [6,7]. In 2016, a study [8] conducted in the Vidarbha region of Central India highlighted the risks associated with roller sports by stating that among various sports, inline skating was identified as the leading cause of orofacial injuries, contributing to 58.4% of reported cases. The most common injuries included lacerations (61.4%) and tooth fractures (25.9%), making inline skating a high-risk activity for dental trauma. Mouthguards, also known as gumshields or sports guards, are essential for preventing dental injuries by protecting teeth, gums, lips, and jaws, reducing injury severity [9,10].

A study found that 13% of orofacial injuries were experienced by children in the age group of 7-12 years during skating, and a properly constructed custom-made mouthguard minimized the common complaints and was seen to be better accepted than the stock and boil-and-bite varieties [5]. In contrast, pre-fabricated and boil-and-bite mouthguards often lack proper fit, thickness, and durability, providing less protection and retention. Ethylene-vinyl acetate is the most popular material for custom-made mouthguards due to its ease of use.

Parents are the primary source of guidance and information for children, often deciding whether their child should wear a mouthguard. Their knowledge and awareness significantly impact its use. Therefore, it is equally important to inform and educate the parents about the importance of wearing mouthguards.

An analysis of existing literature highlights a growing trend in children's participation in skating, which has been associated with a rise in orofacial injuries; however, this increase has not been met with adequate implementation of preventive measures.

While mouthguards are linked to lower injury severity, no conclusive studies prove their effectiveness in skating-related injuries. This underscores the need for further research to assess the prevalence and prevention of such injuries in children aged 8-14 engaged in roller skating.

The study aims to evaluate orofacial injuries in 8- to 14-year-old children playing roller skating with or without a mouthguard.

## Materials and methods

A non-randomized controlled clinical trial was conducted after receiving the Institutional Ethical Committee approval (MUHS/PG-T/E2/09/16/2023) in skating academies of the Nagpur region and the Department of Pediatric and Preventive Dentistry. The study was registered in the Clinical Trials Registry-India (CTRI Registration No: CTRI/2024/05/067414).

Considering a 10% dropout and superiority trial, the estimated sample size was 43 per group [11]. Athletes aged between 8 and 14 years, with erupted first permanent molars, who had completed six months of skating training and were willing to participate in the study but not willing to use a mouthguard, were included. Athletes whose parents did not provide written consent, uncooperative, medically compromised athletes, and those with over-retained teeth, crowded dentition, and undergoing orthodontic treatment were excluded from the study.

After receiving permission from the skating academy, a meeting with athletes and their parents was arranged to explain about mouthguards, its benefits during skating, and about the study.

The athletes whose parents showed willingness to participate in the study, as well as the use of mouthguards by their children during skating, were considered for Group A. The athletes whose parents were willing to participate in the study but not for the use of mouthguard by their children during skating were considered for Group B. Accordingly, informed consent was taken from the parents, and verbal assent was taken from athletes in their vernacular language for participation in the study and allotment was done into Group A - children playing roller skating with a mouthguard of 4 mm thickness, and Group B - children playing roller skating without mouthguard.

**Figure 1:**
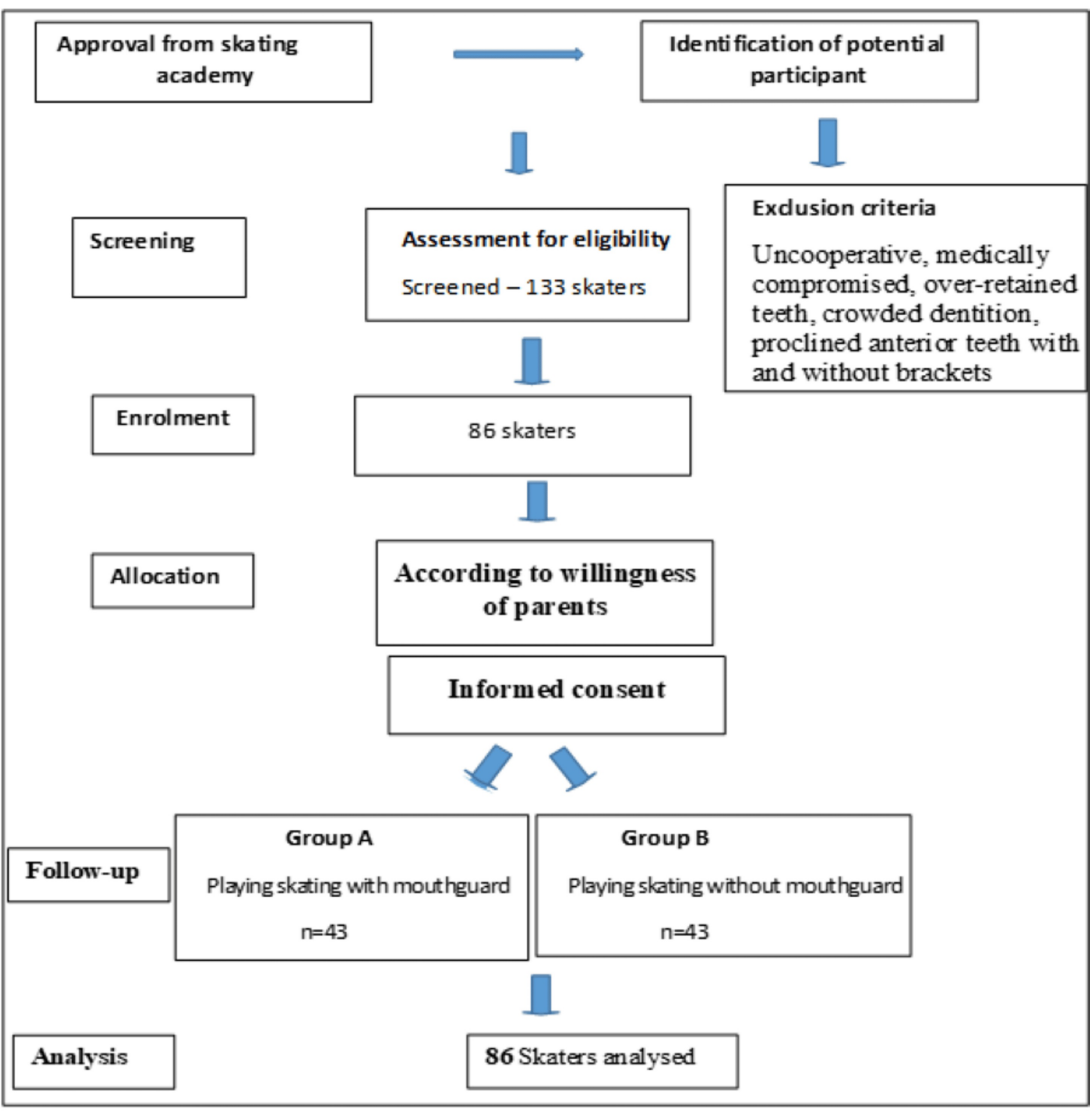
Descriptive flowchart of the study methodology.

A total of 133 children were screened, and 86 athletes were selected based on inclusion and exclusion criteria. A convenience sampling method was used. The descriptive flowchart of the methodology is presented in Figure 1.

The parents of the children from both groups were exposed to the validated questionnaire in their vernacular language at the beginning of the study to evaluate the knowledge of parents towards the use of mouthguards by children while skating (see Appendices A-B).

**Figure 2:**
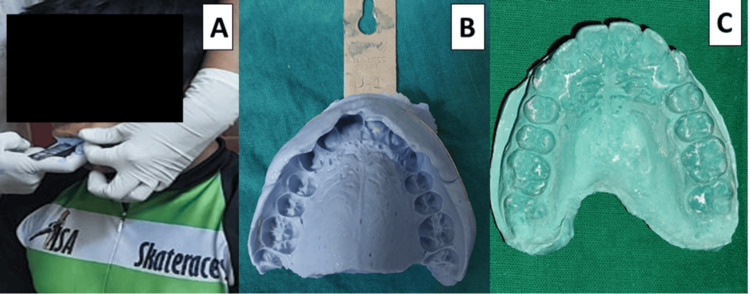
(A) Impression making, (B) maxillary arch impression, and (C) study model.

**Figure 3:**
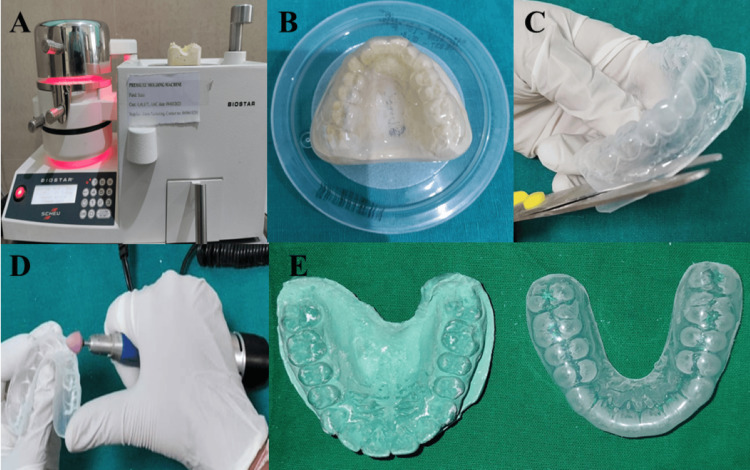
Mouthguard fabrication process. (A) Heating and adaptation of EVA over the cast model; (B) Prepared mouthguard; (C) Removal of excess material; (D) Finishing and polishing; (E) Custom-made mouthguard. EVA: ethylene vinyl acetate

**Figure 4:**
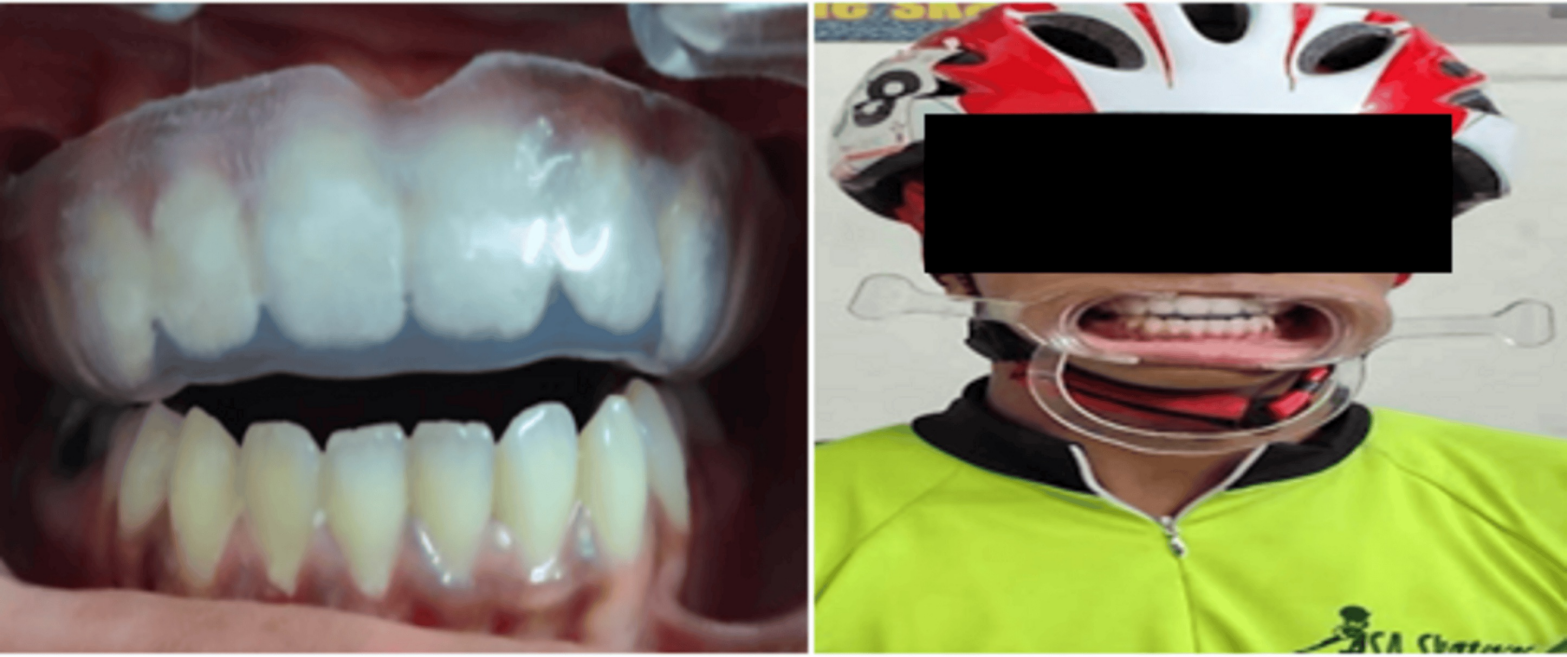
Mouthguard delivery to the athlete.

In Group A, using irreversible alginate dental impression material (Piscium Chromalgin; Piscium Dental Products, Mumbai, India), impressions were made of the maxillary arch, and study models were fabricated with dental stone (Gold Stone, Plaster Type III, Yellow; Asian Chemicals, Gujarat, India) (Figures 2A-2B). A 4 mm-thick mouthguard made of ethylene vinyl acetate (EVA) sheet (Bioplast 4.0 × 125 mm; Scheu-Dental GmbH, Iserlohn, Germany) [12,13] was fabricated using a pressure-molding machine (Biostar; Scheu-Dental GmbH, Iserlohn, Germany) and measured to ensure a uniform 4 mm thickness (Figure 3), as per the guidelines provided by Westerman et al. [14]. After polishing, the mouthguards were evaluated for fit and comfort, and after necessary adjustments mouthguards were delivered to athletes (Figure 4). To ensure compliance, athletes were instructed to wear the mouthguard during skating, and parents were advised to monitor its regular use. Additionally, comprehensive guidance on oral hygiene and mouthguard maintenance was provided to both children and parents at the time of delivery.

Group B athletes participated in the study without using a mouthguard while skating.

Athletes from both groups were observed for one year following the distribution of mouthguards. Parents were asked to report any injuries related to skating. When needed, diagnostic imaging such as intraoral periapical radiograph (IOPA), orthopantomogram (OPG), or cone-beam computed tomography (CBCT) was employed to assess for dental or bone fractures and assist in treatment planning. Throughout the study, athletes were monitored for falls while skating and any associated injuries, including intraoral, extraoral, other bodily harm, and temporomandibular joint (TMJ) pain, both prior to and during the study. Replacement mouthguards were provided if the original ones were lost or damaged.

At the end of one year, the children from Group A were exposed to the validated questionnaire in their vernacular language for evaluating the acceptance of the mouth guard. The validation of both questionnaires was guided by the Content Validity Index (CVI) and the coefficient of reliability. To assess the reliability of the questionnaire, a test-retest reliability study was conducted. Both questionnaires consisted of an eight-item scale with four response options: Strongly Disagree (score 1), Disagree (score 2), Agree (score 3), Strongly Agree (score 4). The maximum possible score for each child was 32, and the minimum score was 8. Based on the obtained scores, mouthguard acceptance by the athletes was evaluated as poor, moderate, good, or excellent.

Statistical analysis

Statistical analysis of the data was done using IBM SPSS Statistics for Windows, Version 26 (Released 2020; IBM Corp., Armonk, New York, United States). The comparison of the frequency of falls in both groups was assessed by the Mann-Whitney test. The comparison of orofacial injuries, fracture of teeth, gingival and oral mucosal injuries in Groups A and B was done by Fisher's exact test. Prevalence of TMJ pain at baseline and follow-up was assessed by the McNemar test.

## Results

Eighty-six athletes (43 in each group) between 8 and 14 years old were included in the study. There were 28 males and 15 female athletes in Group A and 26 males and 17 females in Group B (Table 1).

**Table 1:** Distribution of the study population according to age and sex.

S. No.	Age-wise distribution of children	Groups					
		Group A			Group B		
		Male, n (%)	Female, n (%)	Total, n (%)	Male, n (%)	Female, n (%)	Total, n (%)
1	8 Years	4 (9.3)	4 (9.3)	8 (18.6)	5 (11.6)	3 (6.9)	8 (18.6)
2	9 Years	7 (16.2)	4 (9.3)	11 (25.5)	7 (16.2)	4 (9.3)	11 (25.5)
3	10 Years	5 (11.6)	3 (6.9)	8 (18.6)	3 (6.9)	4 (9.3)	7 (16.2)
4	11 Years	5 (11.5)	3 (6.9)	8 (18.6)	5 (11.6)	3 (6.9)	8 (18.6)
5	12 Years	3 (6.9)	1 (2.3)	4 (9.3)	2 (4.6)	2 (4.6)	4 (9.3)
6	13 Years	2 (4.6)	-	2 (4.6)	2 (4.6)	-	2 (4.6)
7	14 Years	2 (4.6)	-	2 (4.6)	2 (4.6)	1 (2.3)	3 (6.9)
	Total	28 (65.1)	15 (34.8)	43 (100)	26 (60.4)	17 (39.5)	43 (100)
Group A - With Mouthguard, Group B - Without Mouthguard							

Prevalence of fall

The prevalence of falls in both groups was 100% (Table 2). Also, a non-significant difference was noted in the average frequency of falls among the two groups.

**Table 2:** Athletic experience and incidence of falls during skating over a one-year period.

S. No.	Frequency of fall	Groups				p-value (Mann-Whitney test)
		Group A		Group B		
		Number of children	Total number of falls	Number of children	Total number of falls	
1	One time	6	6	5	5	0.962
2	Two times	8	16	7	14	
3	Three times	5	15	8	24	
4	Four times	8	32	9	36	
5	Five times	8	40	6	30	
6	Six times	6	36	4	24	
7	Seven times	2	14	4	28	
Total		43	159	43	161	
Average frequency of fall		3.70 ± 1.81		3.74 ± 1.81		
Group A - With Mouthguard, Group B - Without Mouthguard						

Orofacial and other bodily injuries resulting from a fall

There were 9.3% and 20.9% hard and soft dental tissues injuries in permanent teeth in Groups A and B, respectively, and only 4.6% deciduous teeth in Group B suffered from concussion, which signifies a non-significant difference in the hard and soft dental tissue injuries in permanent teeth as well as deciduous teeth among the two groups (Table 3).

**Table 3:** Hard and soft dental tissue injuries in deciduous and permanent teeth over a one-year period.

Andreasen & Andreasen classification for deciduous teeth	Groups		WHO criteria for permanent teeth	Groups	
	Group A	Group B		Group A	Group B
Enamel fracture (n)	0	0	873.60	1	2
Enamel-dentine fracture(n)	0	0	873.61	0	2
Complicated crown fracture (n)	0	0	873.62	0	1
Crown root fracture (n)	0	0	873.63	0	0
Root fracture (n)	0	0	873.64	0	0
Alveolar fracture (n)	0	0	873.65	0	0
Concussion (n)	0	2	873.66	3	4
Subluxation (n)	0	0	873.67	0	0
Luxation injuries - Lateral luxation/intrusive luxation/extrusive luxation/avulsion) (n)	0	0	873.68	0	0
			873.69	0	0
Total no (%)	0	2 (4.6)		4 (9.3)	9 (20.9)
p-value (Fisher's exact test)	0.494		p-value (Fisher's exact test)	0.228	
Group A - With Mouthguard, Group B - Without Mouthguard					

Injuries were predominantly observed in the maxillary anterior teeth, with no injuries reported in the mandibular region. Group B showed a higher incidence (18.6%) of anterior tooth injuries compared to Group A (4.6%), highlighting the anterior teeth as the most commonly affected in both primary and permanent dentitions (Table 4).

**Table 4:** Region-wise distribution of dental injuries.

Groups	Maxillary (primary and permanent)		Mandibular (primary and permanent)		Total, n (%)
	Anterior, n (%)	Posterior, n (%)	Anterior, n (%)	Posterior, n (%)	
Group A	2 (4.6)	2 (0)	0	0	4 (9.3)
Group B	8 (18.6)	3 (6.9)	0	0	11 (25.5)

None of the subjects in any group reported fracture of the alveolar socket wall, fracture of the alveolar process, or fracture of the base of the jaw. However, 4.7% of athletes in Group B suffered from comminution of the alveolar socket (p=0.494) (Table 5).

**Table 5:** Jaw and facial bone fractures in children due to falls while skating over a one-year period.

Groups	Fracture of the jaw and facial bones				
	Comminution of alveolar socket, n (%)	Fracture of alveolar socket wall, n (%)	Fracture of alveolar process, n (%)	Fracture of base of jaw, n (%)	Total, n (%) (Fisher's exact test)
Group A	0	0	0	0	0
Group B	2 (4.7)	0	0	0	2 (4.6)
p-value	0.494	-	-	-	0.494
Group A - With Mouthguard, Group B - Without Mouthguard					

Among gingival and oral mucosal injuries, 11.6% of athletes in Group A and 25.6% in Group B experienced gingival/oral mucosal lacerations. Contusions were observed in 9.3% of Group A and 14% of Group B, while abrasions occurred in 18.6% of Group B only. These findings show a statistically significant difference between the two groups (p=0.002) (Table 6).

**Table 6:** Intraoral soft tissue injuries (gingival and oral mucosal) in athletes during skating.

S. No.	Groups	Laceration, n (%)	Contusion, n (%)	Abrasion, n (%)	Total, n (%)	p-value
1	Group A	5 (11.6)	4 (9.3)	0	9 (20)	0.002*
2	Group B	11 (25.6)	6 (14)	8 (18.6)	23 (52.6)	
Group A - With Mouthguard, Group B - Without Mouthguard						
Chi-square test; * indicates a significant difference at p≤0.05						

No significant difference was observed in the occurrence of extraoral injury in both groups (51.2% and 62.8% in Group A and Group B, respectively). Chin injuries in both groups were the most common, followed by lip, cheek, and nose injuries in both groups among extraoral injuries (Table 7).

**Table 7:** Extraoral soft tissue injuries in children due to falls while skating over a one-year period. L: laceration; A: abrasion; C: contusion; H: hematoma

Injuries to extraoral soft tissues	Group A, n (%)	Group B, n (%)	p-value (Fisher's exact test)
Nose (L/A/C/H)	2	4	0.708
Lips (L/A/C/H)	7	9	0.904
Chin (L/A/C/H)	9	11	0.970
Cheek (L/A/C/H)	4	3	0.206
Total	22 (52)	27 (62.7)	0.276
Group A - With Mouthguard, Group B - Without Mouthguard			

Thirty athletes in Group A and 33 athletes in Group B showed the leg injury, and 32 athletes in Group A and 35 athletes in Group B experienced the hand injury, demonstrating no statistically significant difference.

TMJ pain analysis

At baseline, TMJ pain was reported by 48.8% in Group A and 51.2% in Group B. After one year, TMJ pain was significantly lower in Group A (23.3%) compared to Group B (62.8%). Intergroup analysis revealed a significant reduction in TMJ pain in Group A compared to Group B after one year (Table 8).

**Table 8:** Prevalence of TMJ pain in athletes playing skating with and without mouthguards: baseline and one-year follow-up. TMJ: temporomandibular joint

Groups	Number of children with TMJ pain				p-value (McNemar test)
	Before the start of study, n (%)		Over one year of study, n (%)		
	Yes	No	Yes	No	
Group A	21 (48.8)	22 (51.2)	10 (23.3)	33 (76.7)	0.035*
Group B	22 (51.1)	21 (48.8)	27 (62.79)	16 (37.2)	0.332
OR (95% CI)	0.91 (0.391-2.122)		0.18 (0.070-0.460)		
p-value (Chi-square test)	0.829		<0.001*		
Group A - With Mouthguard, Group B - Without Mouthguard; * indicates a significant difference at p≤0.05.					

Knowledge of parents towards the use of mouthguards

Parental knowledge of mouthguard use differed significantly between groups (p=0.003, Mann-Whitney test): 11 in Group A and five in Group B had excellent knowledge; 31 in Group A and 27 in Group B had good knowledge; one in Group A and 11 in Group B had moderate knowledge (Table 9).

**Table 9:** Parents’ knowledge and children’s acceptance of mouthguard use during skating.

S. No.	Scores	Knowledge of parents towards the use of mouthguards	Groups		Acceptance of mouthguards by children	Group A, n (%)
			Group A, n (%)	Group B, n (%)		
1	8	Poor	0	0	Poor	0
2	9-16	Moderate	1 (2.3)	11 (25.6)	Moderate	3 (6.97)
3	17-24	Good	31 (72.1)	27 (62.8)	Good	21 (48.8)
4	25-32	Excellent	11 (25.6)	5 (11.6)	Excellent	19 (44)
	p-value: 0.003*					
Group A - With Mouthguard, Group B - Without Mouthguard; * indicates a significant difference at p≤0.05.						

Acceptance of mouthguards by athletes

Based on the questionnaire evaluation of mouthguard acceptance by athletes after one year of use, 19 athletes reported excellent acceptance, 21 reported good acceptance, and three reported moderate acceptance (Table 9).

## Discussion

The observation that children as young as five years are participating in skating highlights the sport's early appeal and developmental potential. Interestingly, skating also attracts children from a diverse range of socioeconomic backgrounds, from low- to high-income groups. This suggests that skating may be a more universally accessible activity, with fewer financial or logistical barriers to entry compared to other sports that often require significant investment in equipment or facilities. A population-based study found that wheel sports account for 35% of fractures among children, making them the second most common cause after ball sports, which are responsible for 42%. Among wheel sports, cycling causes the majority of fractures (63%), while roller and inline skating contribute the remaining 37% [15]. Skating, in particular, is considered a high-risk activity for dental trauma [16]. With the growing popularity of skating both as a recreational and competitive activity, there has been a noticeable increase in related injuries [17].

Several researchers have reported that young individuals who participate in leisure activities such as skateboarding, inline skating (or roller-skating), and bicycling benefit significantly from using appropriate protective equipment, like helmets, knee pads, elbow pads, and wrist guards, which helps to prevent injuries and makes these activities safer [18,19]. Despite the risk of orofacial injuries, face and mouth protection is often overlooked in roller skating. So, a non-randomized controlled trial to assess the effectiveness of mouthguards in preventing such injuries in 8- to 14-year-old skaters over one year was conducted.

Both groups showed slight male predominance, which is consistent with trends seen in pediatric sports activities [20], and had the highest participation at age nine, with balanced age and gender distribution supporting valid group comparisons (Table 1).

Skaters often travel at 20-40 km/h on hard surfaces shared with vehicles and pedestrians [20], where falls are common and can cause injuries ranging from minor to severe. Notably, falls are not always due to individual error; one skater’s fall can trigger others. This supports the present study’s finding that fall frequency is more influenced by situational and environmental factors than by the type of mouthguard or protective gear used (Table 2).

The incidence of hard and soft dental tissue injuries, particularly tooth infarction and concussion, was lower among mouthguard users (9.3%) compared to non-users (23.5%) (Table 3). While this difference was not statistically significant, the trend suggests a potential protective effect. This is supported by a study of Morikawa et al. [21], who demonstrated that mouthguards absorb and disperse shock, reducing vibrational forces to the maxillary anterior teeth, highlighting their role in minimizing sports-related dental trauma.

In the current study, anterior teeth were more commonly affected by trauma than posterior teeth in both permanent and primary dentitions (Table 4), aligning with earlier studies [22,23] that reported sports-related dental injuries primarily involve the maxillary incisors. Müller et al. [23], in their multinational survey, confirmed that anterior teeth are the most commonly impacted by dental trauma, reinforcing the vulnerability of this region in sports-related incidents.

Additionally, none of the participants in either group reported severe skeletal injuries such as fractures of the alveolar socket wall, alveolar process, or the base of the jaw. However, two athletes (4.7%) in Group B sustained comminution of the alveolar socket (Table 5). These observations align with previous literature, which asserts that well-fabricated, custom-fitted mouthguards significantly reduce the incidence of not only soft and hard tissue trauma but also more severe injuries, including concussion and skeletal fractures [18,24].

The incidence of intraoral soft tissue injuries was significantly lower in Group A (27.9%) compared to Group B (69.8%) (p<0.001) (Table 6), supporting the statement of Andreasen and Andreasen classification [25] that mouthguards act as a protective barrier between the hard and soft tissues of the oral cavity, thereby reducing the risk of trauma-related lacerations and contusions.

In contrast, the evaluation of extraoral injuries, primarily skin lacerations, followed by abrasions and soft tissue contusions, revealed no statistically significant difference between the two groups (Table 7). These findings are in line with a study conducted by Lesić et al. [26] reported a high prevalence of facial lacerations among basketball players, underscoring the inevitability of such injuries in high-energy sports and the need for a protective device.

The current study found significantly higher rates of intraoral (69%) and extraoral (62.8%) injuries among non-mouthguard users compared to mouthguard users (12% and 51%, respectively), emphasizing the protective role of mouthguards. These findings align with a previous study [27], which reported that athletes wearing mouthguards experienced lower incidences of tooth fractures (16.9%), soft tissue injuries (25.4%), and non-vital teeth (2.8%) compared to non-wearers, who exhibited tooth fractures in 33.3%, soft tissue injuries in 55.6%, and non-vitality in 11.1%. Notably, no cases of tooth avulsion or luxation were observed in either group.

Conversely, Kalyan [5] reported that 13% of skaters aged 7-12 experienced orofacial injuries when not using mouthguards, whereas no injuries were recorded during a 10-week period of consistent mouthguard use, irrespective of the type used.

There was no significant difference in injury rates to other body parts between groups (Table 8). However, hand injuries were more common than leg injuries in both groups, in align with the study by Christy et al. [28], likely due to reflexive hand movements during falls, a common cause of skating injuries [20].

Maeda et al. [29] highlighted that custom-fitted mouthguards protect the TMJ by maintaining interocclusal space and reducing joint loading. In line with this, the current study found significantly less TMJ pain in mouthguard users after one year, supporting findings by Singarapu et al. [27], who reported that mouthguards reduce TMJ injuries through biomechanical cushioning of impact forces.

Parents in Group A showed significantly greater awareness of mouthguards' protective benefits than Group B (p < 0.001), including support for their use while skating (6 vs. 3; p < 0.001). A significant association was found between parental knowledge and mouthguard use (p=0.003). These findings reflect a stronger perception of the protective benefits of mouthguards among Group A parents, which may explain their greater willingness to support mouthguard use by their children in the current study. The absence of "poor" knowledge scores in both groups suggests a baseline awareness, though disparities in higher knowledge levels highlight the need for targeted educational efforts.

Questionnaire responses after one year indicated favorable acceptance of custom-fitted mouthguards (Table 9), likely due to their comfort and minimal interference with speech and breathing [5,27]. A key strength of this study is the use of a follow-up assessment, which provides insight into the sustained behavioral acceptance of mouthguards over time, adding depth to the findings. However, variations in acceptance may be influenced by factors such as mouthguard thickness and age-related sensitivity to intraoral appliances in children aged 8-14 years.

Limitations

As a non-randomized controlled trial, this study is subject to selection bias. Reliance on self-reported data may introduce recall bias, particularly in children. The focus on 8- to 14-year-old roller skaters limits generalizability to other age groups or sports. Mouthguard compliance was not directly monitored, and external variables like protective gear and environment were not controlled. Additionally, long-term outcomes beyond one year were not assessed.

## Conclusions

Although the frequency of falls was similar between athletes with and without mouthguards, the findings of this study highlight the significant protective benefits of mouthguards in reducing dental hard and soft tissue injuries, gingival and oral mucosal trauma, and TMJ pain among children engaged in roller skating. Although mouthguards did not affect the incidence of extraoral or other bodily injuries, their overall acceptance by young athletes was favorable, indicating that compliance can be enhanced through education and proper customization. Despite limitations such as a non-randomized design, self-reported data, and a narrow age range, the evidence supports the recommendation that coaches, parents, and sporting authorities promote or mandate the use of custom-made mouthguards as standard protective gear in children's roller-skating activities.

## Figures and Tables

**Figure 5:**
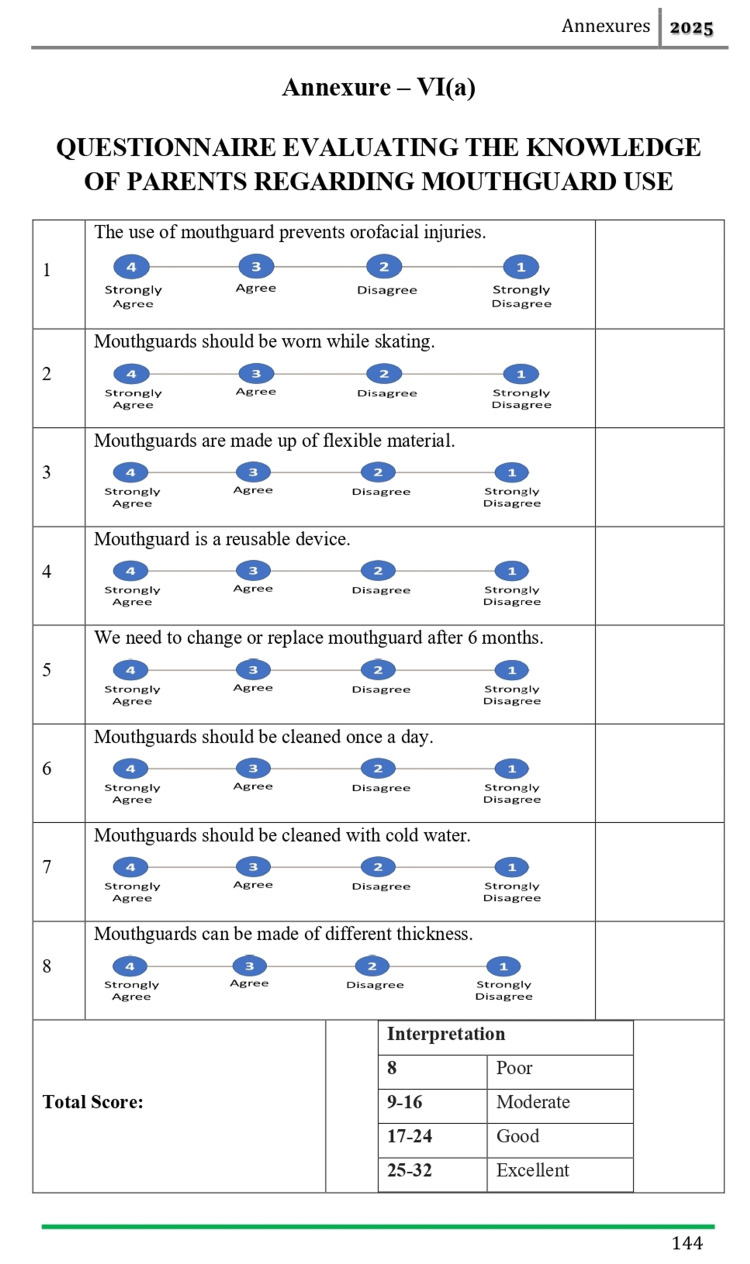
Questionnaire evaluating the knowledge of parents regarding mouthguard use.

**Figure 6:**
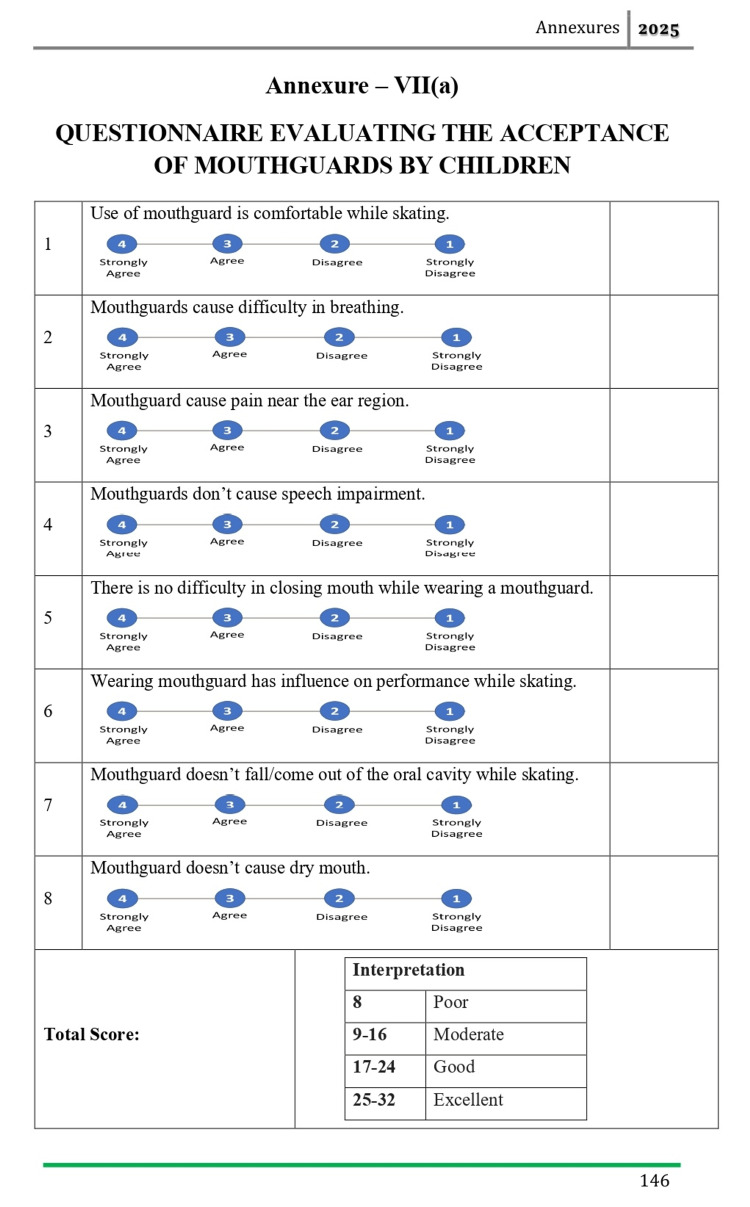
Questionnaire evaluating the acceptance of mouthguards by children.

## Appendices

Appendix A

Appendix B
